# A Case Study of Bluetooth Technology as a Supplemental Tool in Contact Tracing

**DOI:** 10.1007/s41666-021-00112-9

**Published:** 2022-01-19

**Authors:** Ryan Admiraal, Jules Millen, Ankit Patel, Tim Chambers

**Affiliations:** 1 School of Mathematics and Statistics, Victoria University of Wellington, Wellington, New Zealand; 2 Precision Data Science, Wellington, New Zealand; 3 Health, Environment & Infection Research Unit, Department of Public Health, University of Otago, Wellington, New Zealand

**Keywords:** Contact tracing, Bluetooth, Reciprocity, Non-compliance, COVID-19

## Abstract

We present results from a 7-day trial of a Bluetooth-enabled card by the New Zealand Ministry of Health to investigate its usefulness in contact tracing. A comparison of the card with traditional contact tracing, which relies on self-reports of contacts to case investigators, demonstrated significantly higher levels of internal consistency in detected contact events by Bluetooth-enabled cards with 88% of contact events being detected by both cards involved in an interaction as compared to 64% for self-reports of contacts to case investigators. We found no clear evidence of memory recall worsening in reporting contact events that were further removed in time from the date of a case investigation. Roughly 66% of contact events between trial participants that were indicated by cards went unreported to case investigators, simultaneously highlighting the shortcomings of traditional contact tracing and the value of Bluetooth technology in detecting contact events that may otherwise go unreported. At the same time, cards detected only 65% of self-reported contact events, in part due to increasing non-compliance as the study progressed. This would suggest that Bluetooth technology can only be considered as a supplemental tool in contact tracing and not a viable replacement to traditional contact tracing unless measures are introduced to ensure greater compliance.

## Introduction

Contact tracing is a public health tool used to combat the spread of infectious diseases by identifying close personal contacts or shared locations of a case with an infectious disease [1]. It enables health officials to find the source of transmission, identify individuals who potentially may be at risk of having contracted the disease from the source, and prevent future spread of the disease. This information can be used to facilitate the development of intervention and containment strategies, such as treatment or quarantining of infected and at-risk individuals.

Traditional contact tracing begins with notification that someone has an infectious disease. A health official then interviews the infected person, asking them to recall their contacts for an epidemiologically relevant number of days before symptom onset. While this method of contact tracing has been shown to reduce disease transmission in epidemics [2, 3], it is time-consuming, restricted to social contacts known to the case [4, 5], and reliant on frequently imprecise and unreliable human memory [6].

With the rapid spread of COVID-19, the need for accurate and timely contact tracing has become a top priority, and so too has the need for effective methods of collecting and analysing contact tracing data. Existing digital technologies such as Bluetooth and global positioning system (GPS) are increasingly being turned to as means to meet this demand for efficient and reliable automated contact tracing (ACT) [7, 8], with simulation studies suggesting that even modest levels of uptake can be effective [9]. Several simulation studies have examined the effectiveness of ACT based on adoption rates [10, 11], with one finding that an adoption rate as low as 20% of the population could still result in ACT being more effective than traditional contact tracing, largely due to the speed with which contact tracing can be performed [4]. ACT may assume greater importance as COVID-19 variants become more infectious and have shorter incubation periods, both increasing the total number of cases and reducing the time available to prevent the spread of the disease through traditional contact tracing [12]. However, most research on the use of digital technologies in contact tracing has only occurred since the arrival of COVID-19, and there is much that still is not adequately understood about the use of these technologies in contact tracing [13], including the surplus of contact events that would be expected to be detected through the use of ACT [14].

In this paper, we present results from an in vivo trial of Bluetooth technology in assisting with contact tracing in New Zealand. We assess the accuracy of both traditional contact tracing and Bluetooth technology in detecting contact events between individuals. Additionally, we compare the use of Bluetooth technology with traditional contact tracing to highlight the benefits of this technology as a supplemental tool to traditional contact tracing approaches.

## Study Site

The New Zealand Ministry of Health (MoH) commissioned a pilot study from November 9 (Monday) to 15 (Sunday) of 2020 for a Bluetooth Low Energy (BLE) device approximately the size of a credit card (“card” from here on) in the Ngongotah$\bar {\text {a}}$ community in Rotorua City. This community is relatively isolated with a population of approximately 2,500 people. A sample of 1,191 voluntary study participants aged 19 years and older was obtained from those who had their primary residence in Ngongotah$\bar {\text {a}}$ or worked within Ngongotah$\bar {\text {a}}$ Village. (See [15] for more details.) Participants provided informed consent, and the research was approved by the University of Otago Human Ethics Committee (application HE20/010).

## The Card

The card uses a Nordic nRF52832 system on a chip (SoC), which includes a BLE 5.2 capable subsystem and a 32.768 kHz real-time clock crystal oscillator for time and date tracking with a storage capacity of 512 kB of flash memory and 64 kB of RAM. The card housing is based on the Minew BLE Beacon Card C7 and is ultrasonically welded to produce a waterproof seal. A nearly identical device went through a series of tests to assess its robustness (exposure to solvents, three-point bending test, immersion in water, crush testing) which found the device to be robust against the level of expected wear and tear that might reasonably be encountered in everyday use. Prior to deployment, a 2-day test of the card was carried out in a workplace and did not show any hardware or firmware issues with the card [16].

There are a variety of options that could have been considered by the MoH for their device. [17] provides an overview of ACT approaches (including Bluetooth, BLE, WiFi, GPS), focussing primarily on BLE and providing an overview of a variety of data storage, privacy, and security issues and how various countries and technologies/tools have attempted to address these. [17] exclusively considered the use of smartphones in ACT. As we discuss later, BLE signal can be highly variable, and this is further exacerbated when using smartphones which have different hardware, casings, and firmware requiring separate calibration. Additionally, people may not naturally carry smartphones in similar locations on or around the body, leading to increased difficulty in estimating the proximity of two people. The use of common hardware, casings, firmware, and recommended location on the body where the device is carried, as was done in the trial we consider, helps to reduce this variability. Additionally, [17] note that Apple iOS restricts the ability of apps using BLE for location tracking to run in the background, creating problems for iPhone users.

The card stores data locally with the card’s broadcast pseudonym/identifier cryptographically generated and rotated every 15 min as a means to protect the anonymity of card users. Unlike technologies used in Singapore or parts of Europe, this pseudonym is not generated by a central server but rather locally on the card and is included in the device’s advertising packet to other devices. Users can upload their activity logs to a central server which stores pseudonyms, allowing for individuals to be contacted if their pseudonym appears in the list of pseudonyms considered to be at risk from an infected individual based on contact duration and proximity parameters [16].

## Data

All study participants were instructed to wear the card when outside of their homes to detect contact events between card wearers, and data from these contact events were recorded on individual cards. At the end of the trial period, a subset of participants from the main trial were contacted by MoH case investigators to establish contacts that they had over the trial period using a modified version of the MoH case investigation protocol.

### Card Data

The card is designed to be worn on a lanyard around the neck when in environments where people congregate, such as public transport, workplaces, or restaurants. The card has a battery life of approximately 12 months, and no identifiable information is stored on the card itself. Each card both advertises its presence and scans for the presence of other cards. The received signal strength indication (RSSI) in decibel-milliwatts (dBm) over the duration of each encounter with another card is recorded and can be used to estimate the duration of the contact event as well as the proximity of the wearer of the other card.

RSSI is known to be highly variable due to a variety of factors, such as body positioning, antenna beam direction, and multipath interference [18–20]. To address this variability in RSSI when estimating proximity, it is typically the case that raw RSSI values are smoothed using an exponentially weighted moving average (e.g., [21]) or mean (e.g., [22]), median (e.g., [23]), or weighted average of RSSI readings within a given time window (e.g., [21]). The raw or smoothed RSSI values are used to estimate distance, most commonly using a variant of the path loss model for which

$$ \text{RSSI} \propto -20 \log_{10}(\text{distance}), $$

an application of the inverse-square law but on the log-scale and when using experimental measurements taken in free space to calibrate the model [24]. Frequently, a Kalman filter is subsequently applied to further reduce noise in distance estimates (e.g., [21, 23, 25–27]).

The data we use implemented the path loss model to estimate distances from RSSI values but discretised those estimated distances into proximity classes representing distance intervals, as shown in Table 1. Cards recorded interactions in 2-min increments with RSSI readings every 15 s. If an RSSI reading was not obtained continuously for 2 min or was less than − 62 dBm for a given interaction, then it was not stored in the short-term cache memory of the card. Two-minute records were assigned to a proximity class based on the maximum RSSI over the 2-min period. Estimated distances corresponding to maximum RSSI are based on measurements taken for two cards positioned vertically at the same height on tripods in an anechoic chamber [20]. For a given pair of cards, interactions recorded on each card were further aggregated into 2-h blocks for saving in long-term flash memory, resulting in a 2-h record for each card with counts of 2-min blocks at each proximity class.

**Table 1:** Receiver signal strength indication (RSSI) ranges, corresponding estimated distances between cards, and proximity classes

RSSI (dBm)	Distance (m)	Proximity Class
[− 20,− 50]	[0, 1]	0
(− 50,− 56]	(1, 2]	1
(− 56,− 62]	(2, 4]	2

At the end of the pilot study, the interaction data stored on each card was downloaded by the card supplier and supplied to the MoH. Prior to analysis we further aggregated durations for each proximity class from 2-h blocks to daily measures for each unique dyad (or pair) of observing and observed cards in order to produce a dataset that was comparable to case investigation data in terms of the time period for which contact events were recorded.

Card data from only 777 (65.24%) of the 1,191 participants could be analysed, as 261 (21.91%) participants did not return their cards at the conclusion of the trial and 153 (12.85%) cards did not have any recorded interactions for the trial period. There are four likely reasons why a returned card might have recorded no interactions:

1. Participants complied with instructions but did not come into contact with other participants during the study period.
2. Participants did not comply with instructions and failed to wear their card.
3. There was a technical fault in the card firmware, leading to no interaction data being recorded.
4. Data were accidentally deleted by the card supplier in downloading data from individual cards. More recent use of this technology in quarantine facilities suggests that this is a more common problem than originally thought [28].

To ensure all cards used in the dataset had the possibility of observing both sides of an interaction, all cards that appeared as observing cards but never as observed cards, and vice versa, were removed. This resulted in 41 (3.44%) cards being removed from the dataset. Exploratory analysis of the card data showed that the data for the first day (Monday) and last day (Sunday) of the trial had a considerably higher number of interactions compared to the middle 5 days of the trial. Although it is possible that this may be due to a rapid drop in compliance after the first day and then a spike in compliance on the last day, it is more likely that these higher numbers of contact events are due to the gradual roll-out and collection of devices on these days, leading to a number of cards being in close proximity to each other either prior to being distributed to participants (on the first day) or after being collected from participants (on the seventh day). To ensure that data were indicative of expected participant behaviour over an extended time period, the decision was made to remove data for the anomalous first and last day of the trial. This resulted in the removal of a further 63 (5.29%) cards. The final card dataset consisted of 673 cards (56.51% of the original 1,191 cards) and 6,222 recorded day-level contact events.

### Case Investigation Data

At the conclusion of the trial, 158 study participants (13.27% of the original 1,191 participants) were interviewed by MoH case investigators according to a modified case investigation protocol. These interviews occurred November 16–20, the 5-day period immediately following the conclusion of the card trial. Case investigators asked for the date, name of contact, and duration of each contact event. All reported durations were at least 2 min, meaning that these contact events could have been detected by cards if both individuals participated in the trial. Case investigators were told not to record repeated contact events with an individual within the same day, but it was emphasised that contact events with the same individual across multiple days should be recorded as separate contact events.

Case investigations carried out for trial participants differed from typical MoH case investigations in the following ways:

- Case investigators only interviewed participants on one occasion about their contact histories. In practice, case investigators will typically make multiple calls to an individual to produce a complete contact history.
- Case investigators did not ask for additional sources of verification (e.g., bank records, calendars) that would usually be requested. These additional sources are typically only requested to verify locations that a person visited rather than the individuals with whom a person reported coming into contact. Consequently, this would not seem to impact the quality of the person-to-person contact data collected by MoH case investigators.
- The stakes under which these case investigations were carried out were much lower than in “real life” because the person interviewed did not have COVID-19. Consequently, participants may not have had the same motivation to carefully recount their contacts over the preceding week.
- Case investigators were volunteering their time to carry out these case investigations, so they may not have been as motivated as in a real investigation to carefully probe for additional information.
- Case investigators used a bespoke online application to record contact events, which increased the total time required to conduct case investigations. This may have impacted the extent of the investigation.

Of the 158 participants who were interviewed by case investigators, 82 (51.9%) reported contact events only with people who did not participate in the study or could not be identified as a study participant. Case investigation reports by these participants were removed, as there was no means to assess the accuracy of reported interactions involving these participants. Another 11 (6.96%) participants reported contact events with other study participants but either did not return a card, had no card interactions, or either only detected other cards or were only detected by other cards. Although case investigation data provided by these participants could be retained for the purpose of assessing the consistency of case investigation reports by both individuals involved in a contact event, we opted to remove these participants to produce a subpopulation of case investigation participants that was nested within the subpopulation of card participants used in our analyses. This would ensure that data used to assess consistency between case investigation and card contact events matched data to assess the accuracy of case investigation contact events and the accuracy of card contact events. This reduced the dataset to 65 case investigation participants (41.14% of the original 158 case investigation participants) and a total of 362 reported contact events with other trial participants represented in the final card dataset.

## Methods

To assess the accuracy of contact events for the two participants involved in an interaction, both as reported in case investigations and as detected by cards, we looked at measures of reciprocity separately for the two datasets. These reflect the propensities for participants or cards to agree in terms of the occurrence of a contact event. Note that such measures do not actually measure “accuracy” in terms of comparing reported or detected contact events against known interactions. However, in the absence of information on the true (non-)occurrence of contact events, we assume that reported or detected contact events have in fact occurred and internal consistency in reported or detected contact events provides a proxy for the likelihood of accurate registering of the contact events when a case is identified. Agreement between both individuals involved in a contact event signals that the contact event would be registered regardless of who was identified as a case, whereas disagreement calls into question whether the contact event would be recorded, as it would depend on which individual was a case.

To understand the potential benefits of supplementing case investigation data with card data, we examined the consistency between reported contact events in case investigations and interactions detected by cards.

### Reciprocity

To assess the accuracy (or internal consistency) of contact events reported to case investigators, the case investigation data were further reduced to only reported interactions between people who were both participants in case investigations, leading to 90 contact events for 23 participants (14.56% of the original 158 case investigation participants). For these data we estimated reciprocity of reported contact events across the trial period. Each dyad (i.e., pair of participants) for each day was classified as either “mutual” (both participants reported a contact event with each other), “asymmetric” (only one participant reported a contact event with the other participant), or “null” (neither participant reported a contact event with each other).

Reciprocity was estimated using two measures for network data, which are based on a directed adjacency matrix of the contact events. An adjacency matrix $\mathbf {A} = \left \{a_{ij}\right \}_{1 \leq i, j \leq N}$ is an *N* × *N* matrix where *N* denotes the number of nodes (i.e., participants in the case of the case investigation data, cards in the case of the card data) and

$$ \begin{array}{@{}rcl@{}} a_{ij} &=& \left\{\begin{array}{ll} 1, & \text{there is an edge directed from node \textit{i} to node \textit{j} (and $i \neq $j),} \\ 0, & \text{otherwise}. \end{array}\right. \end{array} $$

Here, an edge represents a contact event, so *a*_*ij*_ = 1 indicates that participant *i* reports a contact event with participant *j*.

The first measure of reciprocity we considered is the traditional measure of reciprocity *r*, where

$$ \begin{array}{@{}rcl@{}} r &=& \frac{\|\mathbf{A} \odot \mathbf{A}^{\prime}\|}{\|\mathbf{A}\|} \ = \ \frac{2\vert\text{mutual dyads}\vert}{2\vert\text{mutual dyads}\vert + \vert\text{asymmetric dyads}\vert}. \end{array} $$

Here, $\mathbf {A}^{\prime }$ is the transpose of **A**, ⊙ denotes elementwise product, ∥**X**∥ denotes the sum of elements of matrix **X**, |*x*| denotes the cardinality of set *x*, and mutual and asymmetric dyads are defined as

$$ \begin{array}{@{}rcl@{}} \text{mutual dyad} &\equiv& \text{unordered pair of nodes } (i,j) \text { where } a_{ij} = a_{ji} = 1 \\ \text{asymmetric dyad} &\equiv& \text{unordered pair of nodes } (i,j) \text { where } a_{ij} \neq a_{ji}. \end{array} $$

This measure is simply the empirical proportion of reported contact events that are reciprocated (i.e., if *a*_*ij*_ = 1, then *a*_*ji*_ = 1). While the calculation of *r* is straightforward and allows for the estimation of variability associated with *r* using a binomial distribution, *r* does not account for information on the level of reciprocity that would be anticipated for a network with *N* nodes and $\|\mathbf {A}\| = {\sum }_{i \neq j}a_{ij}$ randomly distributed edges (i.e., random contacts for the 90 reported contact events among the 23 participants considered in this analysis). This means that the same value of *r* for two networks with different numbers of nodes and/or edges need not indicate the same level of reciprocity relative to what might be expected under a random distribution of edges for the two networks.

The second measure of reciprocity we considered is a measure proposed by [29], denoted by *ρ*. This measure is estimated by

$$ \begin{array}{@{}rcl@{}} \widehat{\rho} &=& \frac{\|\left( \mathbf{A} - \overline{a}\right) \odot \left( \mathbf{A}^{\prime} - \overline{a}\right)\|}{\|\left( \mathbf{A} - \overline{a}\right) \odot \left( \mathbf{A} - \overline{a}\right)\|} \ = \ \frac{r - \overline{a}}{1 - \overline{a}}, \end{array} $$

where

$$ \begin{array}{@{}rcl@{}} \overline{a} &=& \frac{\|\mathbf{A}\|}{N(N - 1)} \ = \ \frac{{\sum}_{i \neq j}a_{ij}}{N(N - 1)} \end{array} $$

denotes the network density (i.e., observed number of edges relative to the maximum possible number of edges) and *r* is the previously described traditional measure of reciprocity. This measure is similar to the intraclass correlation coefficient based on corresponding cells *a*_*ij*_ and *a*_*ji*_ of the adjacency matrix **A**, ranging from $-\frac {\overline {a}}{1 - \overline {a}}$ when *r* = 0 (anti-reciprocity) to 1 when *r* = 1 (reciprocity) with a value of $\widehat {\rho } = 0$ corresponding to a reciprocity (i.e., a random distribution of edges). This measure converges to the traditional measure of reciprocity as the network density tends toward 0 (i.e., $\widehat {\rho } \longrightarrow r$ as $\overline {a} \longrightarrow 0$). Corresponding to $\widehat {\rho }$, the expected standard deviation of the estimator can be estimated using the jackknife, producing

$$ \begin{array}{@{}rcl@{}} \widehat{\sigma}_{\rho} &=& \sqrt{\sum\limits_{i < j}\left( \widehat{\rho}_{-(ij)} - \widehat{\rho}\right)^{2}}, \end{array} $$

where $\widehat {\rho }_{-(ij)}$ denotes $\widehat {\rho }$ but for a modified adjacency matrix where *a*_*ij*_ = *a*_*ji*_ = 0 [30].

To try to account for cases where people may have misreported the date of a contact event to a case investigator, a second analysis was carried out where unreciprocated reported contact events were reassigned to the day before or day after if

- they did not report a contact event with the same person for either the day before or the day after and
- moving the contact event to the day before or after led to a reciprocated contact event.

Of the 90 interactions reported over the trial period for the 23 case investigation participants considered in this analysis, this led to only 3 (3.333%) interactions being reassigned. This would seem to indicate that any significant recall bias was unlikely to be due to misreporting the day of a contact event, and results from this second analysis are not presented here.

The day-aggregated card data were analysed for internal consistency in a similar manner to the case investigation data but for all 673 participants for whom card data were available and excluding the first and last days of the trial.

Finally, to compare trends in reciprocity over time for the card data and case investigation data, we fit the nested polynomial logistic regression models for *r* shown in Table 2. Likelihood ratio tests allowed us to assess whether trends in the log-odds of *r* differed (in terms of both intercept and slope) between the card and case investigation data as well as whether a degree 1 or 2 polynomial best fit the trend over time. (Here, the “dataset” variable is a dummy variable indicating whether the reciprocity value corresponds to the card dataset or case investigation dataset, and “day” is a numeric variable ranging from 1 to 7.)

**Table 2:** Polynomial logistic regression models fit to *r*

Model	Independent variables
1	$\log \left (\frac {r}{1 - r}\right ) \sim \text {dataset} + \text {day}$
2	$\log \left (\frac {r}{1 - r}\right ) \sim \text {dataset} + \text {day} + \text {day}^{2}$
3	$\log \left (\frac {r}{1 - r}\right ) \sim \text {dataset} + \text {day} + \text {day}^{2} + \text {dataset} \times \text {day} + \text {dataset} \times \text {day}^{2}$

### Consistency between Case Investigation and Card Data

We additionally assessed the level of consistency between the case investigation and card data for contact events to establish the degree to which inclusion of Bluetooth technology in contact tracing may lead to identification of additional contact events. We did this by producing confusion matrices of contact events indicated by the card data and reported in case investigations, examining the level of concordance in reported (by participants) and detected (by cards) contact events, percentage of contact events reported by participants that were not detected by cards, and percentage of contact events detected by cards that were not reported by participants. This meant restricting analysis to the 65 participants in case investigations who returned their cards, had interactions recorded on their cards and were detected by other cards, and reported contact events with other people in the card trial.

Case investigations are meant to focus on identification of “close” contacts. At the time of the study, [31] defined a close contact as a “face-to-face contact in any setting within two metres of a case for 15 minutes or more.” Cards may detect other cards at distances in excess of 2 m. In particular, proximity class 2 is expected to correspond to distances between 2 and 4 m in a controlled setting. Consequently, in assessing the consistency between case investigation and card contact events, we might initially focus on card interactions where the duration of contact is at least 15 min at proximity classes 0 or 1. However, case investigation participants were not apprised of the MoH definition of “close” contact, and some reported interactions for as short as 2 min in duration. Additionally, as noted previously, a variety of factors can impact Bluetooth RSSI, complicating the relationship between RSSI and proximity. For instance, a recent study by [14] in which they examined the relationship between RSSI and proximity for real-world scenarios found RSSI to be similar for those walking directly next to each other and those walking approximately 2 metres apart in a supermarket setting, while RSSI actually *increased* with distance in a train carriage and home setting. Considering this unclear relationship between RSSI and proximity, we separately evaluated the consistency of reported contact events in case investigations with detected contact events of any non-zero duration at proximity class 0, combined proximity classes 0 and 1, and combined proximity classes 0, 1, and 2.

### Statistical Software

All statistical analyses were carried out using R [32]. The “dplyr” package was used for general data preparation and transformation [33], the “lubridate” package was used to reformat date and time data [34], and the “network” [35, 36] and “sna” [37] packages were used for network analysis.

## Results

### Reciprocity

The internal consistency of day-level contact events showed that, for the card data, 87.72% (95% confidence interval: (86.91%, 88.54%)) of reported contact events over the second to sixth days of the trial period were reciprocated. This was significantly lower for the case investigation data with only 64.44% (54.55%, 74.33%) of reported contact events over the full trial period being reciprocated. Table 3 shows the reciprocity measures *r* and $\widehat {\rho }$ by day for the card data and case investigation data. Note that the network density ranges from 0.0013 to 0.0129 for the card data and 0.0026 to 0.0038 for the case investigation data. These low network densities explain the high degree of similarity between *r* and $\widehat {\rho }$.

**Table 3:** Reciprocity by day for traditional (*r*) and Garlaschelli and Loffredo ($\widehat {\rho }$) measures

Data	Day	$r \ \left (\widehat {\sigma }_{r}\right )$	$\widehat {\rho } \left (\widehat {\sigma }_{\rho }\right )$	*n*
Card	Nov 10	0.8771 (0.0070)	0.8766 (0.0149)	2,214
	Nov 11	0.8616 (0.0092)	0.8613 (0.0188)	1,395
	Nov 12	0.8527 (0.0115)	0.8524 (0.0227)	957
	Nov 13	0.8960 (0.0099)	0.8958 (0.0227)	942
	Nov 14	0.9160 (0.0104)	0.9159 (0.0260)	714
Case Investigation	Nov 9	0.7500 (0.1083)	0.7490 (0.2014)	16
	Nov 10	0.4615 (0.1383)	0.4599 (0.2692)	13
	Nov 11	0.6667 (0.1361)	0.6657 (0.2553)	12
	Nov 12	0.6154 (0.1349)	0.6142 (0.2510)	13
	Nov 13	0.7692 (0.1169)	0.7685 (0.2252)	13
	Nov 14	0.5455 (0.1501)	0.5442 (0.2919)	11
	Nov 15	0.6667 (0.1361)	0.6657 (0.2553)	12

These are presented for the card data and case investigation data

**Fig. 1:**
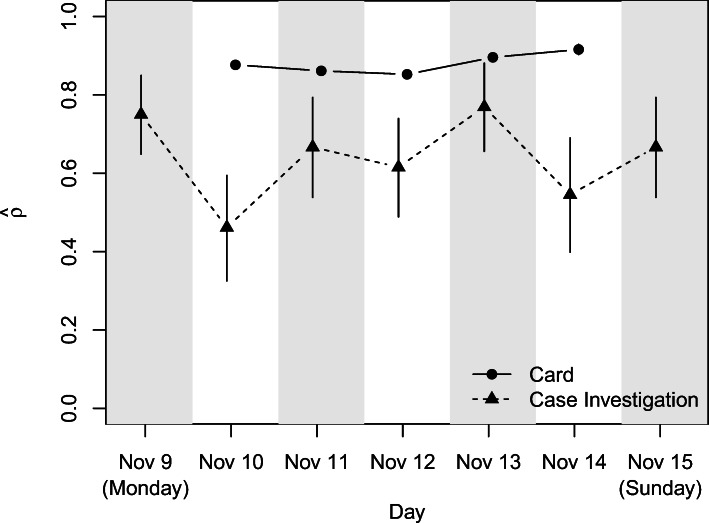
Daily measures of reciprocity using the Garlaschelli and Loffredo measure of reciprocity $\widehat {\rho }$ for card and case investigation data contact events. Vertical lines represent the estimated standard deviation $\widehat {\sigma }_{\rho }$

Figure 1 shows $\widehat {\rho }$, along with corresponding expected standard deviation $\widehat {\sigma }_{\rho }$, for each day for both the card and case investigation data. Reciprocity for contact events indicated by cards appears to stay relatively consistent over days 2 to 6 (November 10–14) of the study. We would anticipate that the number of cards developing defective firmware over such a short period of time would be relatively low, so this consistency is not that surprising. There is significantly more variability in measures of reciprocity for the case investigation data, which can be partially attributed to the small sample sizes on which estimates for the case investigation data are based for each of the 7 days. We might expect memory recall to be worse when reporting on contact events that are more distant in time, meaning that we would likely expect to see an increasing trend in reciprocity over time. There is no clearly discernible increasing (or decreasing) trend, however, failing to indicate any clear change in memory recall over the span of the study for case investigation reports.

**Fig. 2:**
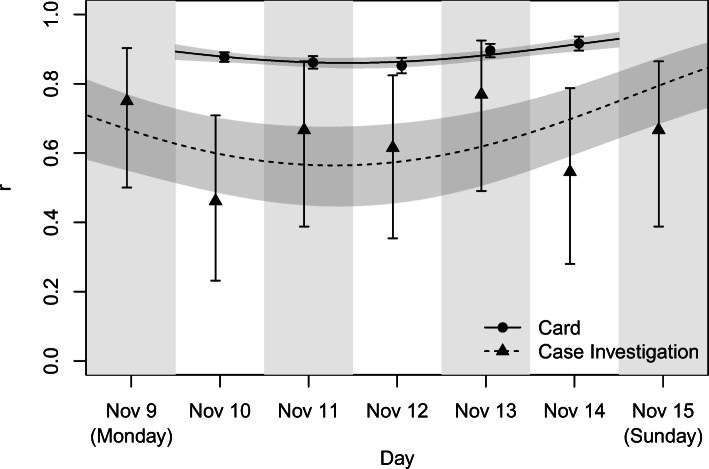
Daily measures of reciprocity using the traditional measure of reciprocity *r* for card and case investigation data contact events. Wald (for card data) and Agresti-Coull (for case investigation data) 95% confidence intervals for *r* are shown as well as logistic regression predicted values (as trend lines) and corresponding 95% confidence intervals (as shaded regions)

Modelling *r* using polynomial logistic regression, we obtain likelihood ratio tests for the nested models as presented in Table 4. These show evidence of a significant improvement in model fit by including a quadratic term for time (*G*^2^ = 12.11, df = 1, *p* = 5.016 × 10^− 4^) but not by additionally considering a dataset-time interaction (*G*^2^ = 1.856, df = 2, *p* = 0.3954). Thus, Model 2 is the preferred model, and summary output for this model is as shown in Table 5. Not surprisingly, a Wald test indicates that the odds of a contact event detected by a card being reciprocated is estimated to be 4.748 (3.022, 7.462) times higher than that of a contact event reported in case investigations, a highly significant difference (*z* = 6.755, *p* = 1.433 × 10^− 11^). Additionally, the quadratic term is highly significant and positive (*z* = 3.448, *p* = 5.655 × 10^− 4^), producing higher estimated reciprocity at time points at the extremes of the study period, as shown in Fig. 2. This is inconsistent with memory recall changing over time for the case investigation data.

**Table 4:** Likelihood ratio tests of Model 1 (linear in time) with Model 2 (quadratic in time) and Model 2 with Model 3 (quadratic in time, interaction with dataset)

Model	Resid. Df	Resid. Dev	Df	Deviance	Pr(> Chi)
1	9	19.97			
2	8	7.860	1	12.11	0.0005016
3	6	6.004	2	1.856	0.3954

**Table 5:** Logistic regression of *r* on the dataset (card or case investigation) and time modeled as quadratic (i.e., $\log \left (\frac {r}{1 - r}\right ) \sim \text {dataset} + \text {day} + \text {day}^{2}$)

	Estimate	Std. Error	z value	Pr(> |*z*|)
Intercept	0.5525	0.2233	2.474	0.01337
dataset(card)	1.558	0.2306	6.755	1.433e-11
day	0.6654	0.1913	3.479	0.0005025
day^2^	0.8525	0.2473	3.448	0.0005655

### Consistency between Case Investigation and Card Data

Confusion matrices showing the level of agreement between contact events reported in case investigations and those detected by cards for proximity class 0, combined proximity classes 0 and 1, and combined proximity classes 0, 1, and 2 are as presented in Table 6. These show that cards detected 31.08% of reported case investigation contact events when using interactions detected at proximity class 0. This increased to 51% when using combined proximity classes 0 and 1 (card distances that should be roughly equivalent to the MoH standard of a close contact at the time of the study), and further increased to 64.54% when using combined proximity classes 0, 1, and 2 (i.e., any detection of another card). The percentage of contact events recorded by cards that were not reported in case investigations was 62.68% when only considering proximity class 0 and increased slightly to 66.18% when considering all three proximity classes.

**Table 6:** Confusion matrices for contact events detected by cards and reported in case investigations over the period November 10–14 for (a) proximity class 0, (b) combined proximity classes 0 and 1, and (c) combined proximity classes 0, 1, and 2

**(a)**	**Proximity Class**	Case Investigation	
	**0**	No Contact	Contact
Card	No Contact	238,818	173
	Contact	131	78
**(b)**	**Proximity Class**	Case Investigation	
	**0 and 1**	No Contact	Contact
Card	No Contact	238,727	123
	Contact	222	128
**(c)**	**Proximity Class**	Case Investigation	
	**0, 1, and 2**	No Contact	Contact
Card	No Contact	238,632	89
	Contact	317	162

**Fig. 3:**
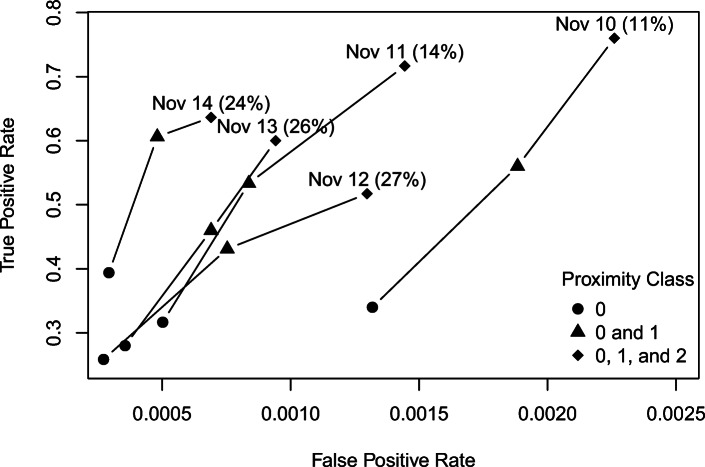
True positive rates and false positive rates by day and proximity class for the ability of cards to detect contact events reported in case investigations. The percentage of individuals reporting contact events to case investigators who had no recorded interactions on their cards is shown in parentheses next to each date

**Fig. 4:**
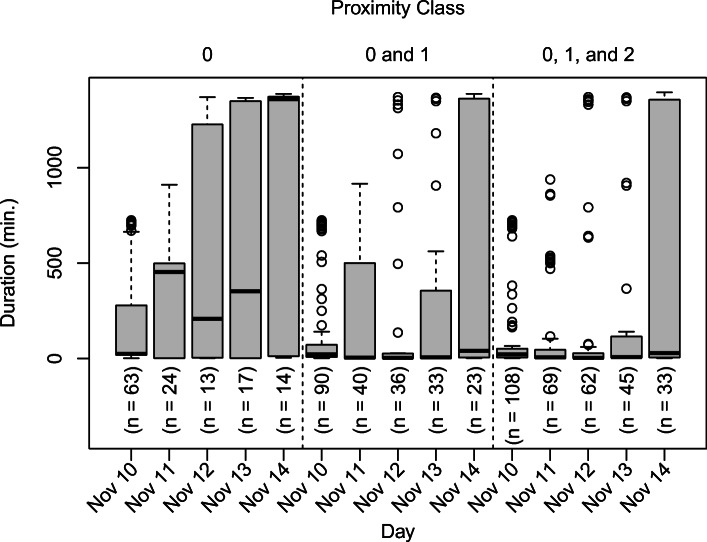
Recorded durations at given proximity classes for instances where cards indicated contact events but these were not reported to case investigators

To assess the ability of cards to accurately detect contact events reported in case investigations, we treat case investigation contact events as the ground truth. In this case, we can represent a confusion matrix by its true positive rate (i.e., proportion of contact events reported in case investigations that are detected by cards) and false positive rate (i.e., proportion of unreported contact events in case investigations that are indicated as contact events by cards). Figure 3 shows true positive rates and false positive rates for each day and for contact events indicated by interactions at proximity class 0, combined proximity classes 0 and 1, and combined proximity classes 0, 1, and 2. (Note that Fig. 3 simply presents partial receiver-operating characteristic curves for different thresholds of defining contact events for the card data, eliminating the non-informative cases where the false positive rate and true positive rate are both 0 or 1.) Card data detected contact events reported to case investigators at higher rates for days 2 (November 10) and 3 (November 11) of the trial than later days when considering interactions detected by cards at combined proximity classes 0, 1, and 2. This can likely be partially attributed to seemingly increased participant non-compliance in wearing cards as the trial progressed. Of those individuals who reported contact events to case investigators, less than 15% had no recorded interactions on their cards for either November 10 or 11. This increased to roughly 25% for each of the remaining days with higher percentages associated with lower true positive rates. At the same time, if we examine durations recorded for those cases where a card indicated a contact event but this went unreported to case investigators, as shown in Fig. 4, we observe increased incidence of unrealistically high durations later in the trial. For instance, for November 10–11 the maximum recorded duration at proximity class 0 for any card for which a contact event was not reported to case investigators was approximately 15.5 h. By contrast, on November 12, 13, and 14 there are 5 (38.46%), 6 (35.29%), and 9 (64.29%) cases of such cards recording interactions of durations of more than 22 h at proximity class 0. These would also likely indicate non-compliance by members of the same household who failed to wear their cards and left them in close proximity to each other.

## Conclusions

The analysis of reciprocity of contact events reported in case investigations highlights the inherent shortcomings of a contact tracing system wholly dependent on traditional contact tracing. Over the course of the study, 35.56% (25.67%, 45.45%) of reported contact events were reported by only one of the two people involved in an interaction with daily rates fluctuating from 23.08 to 53.85%. This would suggest that, if only traditional contact tracing is used, a high proportion of contacts could go unreported, depending on the person who is a case. Although it is possible that participants may have over reported contacts, meaning that true contact events would be picked up regardless of who was the case but false positives may also occur, it is more likely that under reporting occurred, leading to instances of both participants failing to report a contact event. Under reporting is expected to be more likely because both study participants and case investigators were aware that case investigations were not carried out for bona fide cases and so would not have felt the same urgency to ensure to remember all contacts, and the nature of the case investigation would not suggest any clear social desirability to inflate the number of contacts. Additionally, card data show that more than 60% of interactions detected by cards were not reported in case investigations with many of these corresponding to durations that would be consistent with a genuine contact event (see Fig. 4). Consequently, we would expect these estimates of percentages of cases that could potentially go unreported in case investigations to be conservative.

Cards show significantly greater internal consistency than case investigation data in detection (or reporting) of contact events with only 12.28% (11.46%, 13.09%) of contact events detected by only one of the two cards involved and daily rates staying relatively stable, ranging from 6.83 to 15.08%. In spite of this higher internal consistency, it is important to emphasise that neither case investigation data nor card data represent the ground truth when it comes to contact events. Misreporting of contact events to case investigators certainly happens, and previous research has estimated the true coverage of the manual contact tracing process ranges from 61 to 80% of a person’s social network [4, 5]. At the same time, cards do not always agree on whether an interaction occurred, as evidenced by measures of reciprocity for both the case investigation data and the card data being well below 1 for both *r* and $\widehat {\rho }$. Additionally, cards will not detect genuine interactions if not worn, leading to either no detected interactions or unrealistically long interactions if placed near another card. Even when worn, Bluetooth can pass through solid barriers (such as walls) that effectively block any risk of disease transmission, and cards may detect each other at distances for which two individuals have no real risk of transmitting a particular disease. Variability in signal strength can lead to both false positives (i.e., detection of contact events that do not exist) and false negatives (i.e., failure to detect legitimate contact events) [20].

If we assume that case investigation data represent the ground truth, then the cards were able to accurately detect from 51.72 to 76% of contact events over the duration of the study when using detected interactions at any proximity class (see Fig. 3). These percentages are inversely related to the percentages of individuals whose cards recorded no interactions on a given day, highlighting that the suitability of Bluetooth technology as a replacement for traditional contact tracing is dependent on compliance in wearing the card. Figure 3 suggests significantly lower compliance already by November 12 (day 4 of the study) with an approximate doubling in the percentage of cards with no recorded interactions for those reporting contact events to case investigators. Meanwhile, Fig. 4 shows a significant spike in cards detecting unusually long interactions at proximity class 0 for those cases of no contact event being reported to case investigators, with more than 35% of cards recording a duration of at least 22 h at proximity class 0. These also likely correspond to incidents of non-compliance with household members leaving cards at home. Compliance is a major consideration in the application of digital contact tracing technologies with issues of privacy, COVID fatigue and misinformation substantially impacting uptake [38]. However, some countries have implemented a mandate on these technologies. For example, New Zealand now requires citizens to either use the NZ COVID Tracer app or manually provide their details upon entering essential services under lockdown conditions [39]. While such measures may allow for better detection of shared locations with a case, the New Zealand mandate does not require the activation of Bluetooth, and there is no way to ensure that there would be compliance in a range of settings, such as non-essential services or while in public. Given these issues with compliance as well as difficulties in establishing accurate estimates of proximity from RSSI across a range of settings [14], Bluetooth technology cannot presently be considered to be suitable as a replacement for but rather a supplemental tool to traditional contact tracing.

Finally, we note that it is necessarily the case that cards will accurately detect increasing (or, at worst, the same) percentages of reported contact events as the distance of the interaction is expanded (i.e., consider additional proximity classes corresponding to further distances). However, this comes with the trade-off of increasing (or, at worst, the same) rates of false positives (i.e., detected contact events that are not at risk of transmitting the disease). The costs (both economic and social) associated with false positives and false negatives will vary depending on the disease in question, and statistical modelling can be used to determine an optimal distance/proximity class and/or duration to define a contact event based on these costs, represented in a loss function. However, this requires that the ground truth be known, which is difficult to ascertain outside of a highly controlled setting. Further, with diseases such as COVID-19 where the emergence of variants may quickly lead to different transmission dynamics and costs associated with false positives and negatives, such approaches may require frequent model updating.
